# Feasibility of leveraging menstrual cycle tracking apps for preconception research recruitment

**DOI:** 10.3389/frph.2022.981878

**Published:** 2022-09-30

**Authors:** Anne Marie Z. Jukic, Hannah R. Jahnke, Nathaniel MacNell, Danielle Bradley, Shannon M. Malloy, Donna D. Baird

**Affiliations:** ^1^ Epidemiology Branch, National Institute of Environmental Health Sciences, Durham, NC, United States; ^2^ Public Health Sciences, Social / Scientific Systems, Inc., a DLH Company, Durham, NC, United States; ^3^ Department of Clinical Services and Evidence, Ovia Health, Boston, MA, United States

**Keywords:** menstrual cycle, tracking app, mobile application, digital health, time to pregnancy, fertility

## Abstract

**Background:**

Mobile applications (apps) present a new opportunity to study menstrual cycles and time to pregnancy. Understanding the characteristics of cycle tracking app users is important to evaluate the feasibility of recruiting participants for preconception research.

**Methods:**

Users of a cycle tracking smartphone app, Ovia Fertility, aged 18 or older in the U.S. were randomly invited *via* email to complete a “fertility research” questionnaire that included demographic and reproductive characteristics. Among those attempting pregnancy without medical assistance, attempt duration, factors influencing pregnancy planning, health history and behaviors while attempting to conceive were queried. Respondents could choose to enter a raffle for a $50 gift card.

**Results:**

Initially, 639 people responded to the demographics portion of the survey representing 49 states and Washington DC. Of these, 344 (54%) were trying to conceive and of those, 297 (86%) were not using medical treatments. Of those not trying to conceive, 12% reported that they planned to start in the next 3 months. Most participants were ages 26–35 (63%), of White race (70%), reported non-Hispanic ethnicity (87%), had at least a bachelor's degree (56%) and an income between $50,000 and $200,000 (58%). One-third were of recommended BMI (35%), 24% overweight, and 41% obese. Most participants reported no fertility-related health conditions (58%). Forty-eight participants (17%) had been trying to conceive for 1 month or less, 88 (31%) had been trying for 2 months or less, and 122 (43%) for 3 months or less. Interruptions in pregnancy attempts were common, 31% reported periods without intercourse. Of those attempting pregnancy, 47% of partners completed their own questionnaire.

**Conclusion:**

This first-of its-kind analysis describes users of a cycle-tracking smartphone app who could be eligible for recruitment to a prospective time-to-pregnancy study. Survey respondents were diverse with respect to geographic location, BMI, and income. However, special recruitment efforts will be needed to recruit participants and partners who identify as other than non-Hispanic White. Participants with fertility-related conditions are not overly represented among app users who are trying to conceive. Targeting and pre-enrolling app users who are planning to begin a pregnancy attempt in the next 3 months may be an advantage of app-based recruitment.

## Introduction

Mobile applications (apps) for tracking menstrual cycles and aiding conception are increasing in popularity. Using an app can help people time their intercourse in relation to the fertile window of the menstrual cycle to either increase the probability of conception (1) or to avoid conception (2). Data collection through the apps is fast, convenient, and helps users understand their own menstrual cycle patterns (3). This increase in popularity means that millions of people contribute to large databases of menstrual cycles and time to pregnancy. These data present a new opportunity to study menstrual cycles and time to pregnancy with detailed data and sample sizes that have never been available before in this research field (4). Before embarking on a large prospective study of menstrual cycles or time to pregnancy, however, it is important to understand the underlying population using these apps. While cycle tracking apps may provide a diverse database of potential study participants for research purposes, this has just begun to be empirically examined; the demographic, behavioral, and reproductive characteristics of app users need further investigation (3).

To plan for app-based recruitment to a time-to-pregnancy study, it is important to characterize app users’ decisions about planning to conceive, the distribution of ongoing attempt timing, and their behaviors while trying to conceive. There is also reason to believe that users of cycle tracking apps may be more likely to have subfertility challenges and are therefore more predisposed to tracking their cycle more closely with the assistance of a digital tool. These individuals may therefore be overrepresented in app-based recruitment studies, thus understanding the prevalence of subfertility and related conditions in these userbases is crucial. Finally, it is important to gauge app users’ interest in research participation, their willingness to provide data beyond that routinely collected by the app, and their partner's interest in study participation.

To address these literature gaps, this pilot study investigates the characteristics of users of a menstrual cycle tracking app called Ovia Fertility who agreed to participate in an online research study. The objectives of this study were to: (1) describe the demographic characteristics of all questionnaire respondents recruited through Ovia Fertility and compare with the Ovia Fertility userbase, (2) estimate the proportion of individuals using the app who plan to start a pregnancy attempt in the next 3 months and could therefore be eligible for enrollment in a time-to-pregnancy study; and (3) estimate the proportion of users currently attempting pregnancy without medical interventions who would be immediately eligible for a time-to-pregnancy study. Additionally, among those who were currently attempting to conceive without medical interventions, we aimed to: (4) assess the diversity of their demographic and reproductive characteristics and describe characteristics of their current pregnancy attempt; (5) describe the factors that influence app users’ decisions around pregnancy planning; and (6) assess willingness of partners of active app users to participate in an app-based research study. The data provided here will inform study design, questionnaire development, sample size calculations, approximate study recruitment timelines, and provide preliminary data on diversity and representativeness.

## Materials and methods

The Ovia Fertility app is a free downloadable menstrual cycle tracking app available on both iOS and Android operating systems (https://info.oviahealth.com/enroll). Data that can be recorded by the user include historic and current menstrual period duration and symptom data such as basal body temperature, cervical fluid, physical and emotional symptoms, medications, diet, and exercise. The app is customizable so users can track data points that are most interesting to them, such as symptoms, mood, exercise, sex, or nutrition. Summary data are provided to the user, such as average cycle length, period length, and most frequent symptoms.

To investigate the demographics of Ovia Fertility users likely to participate in research about time-to-pregnancy or preconception studies, we used the Ovia Fertility user database to send emails to a subset of Ovia Fertility users, randomly selected from all users aged 18 or older in the United States. Emails were sent between August 21–25, 2020 and September 17–October 7, 2020 to 90,725 users. The email described a research study of “women's reproductive health” and “factors that influence fertility” and contained a unique link to an electronic questionnaire (Supplementary Figure S1). The number of emails sent, the number of emails opened, and the number of unique link clicks was tracked. Our aim was not to recruit every person who received an email, but to obtain at least 250 responses. Respondents who were willing to provide their contact information were entered into a drawing for one of four $50 gift cards.

**Figure 1:**
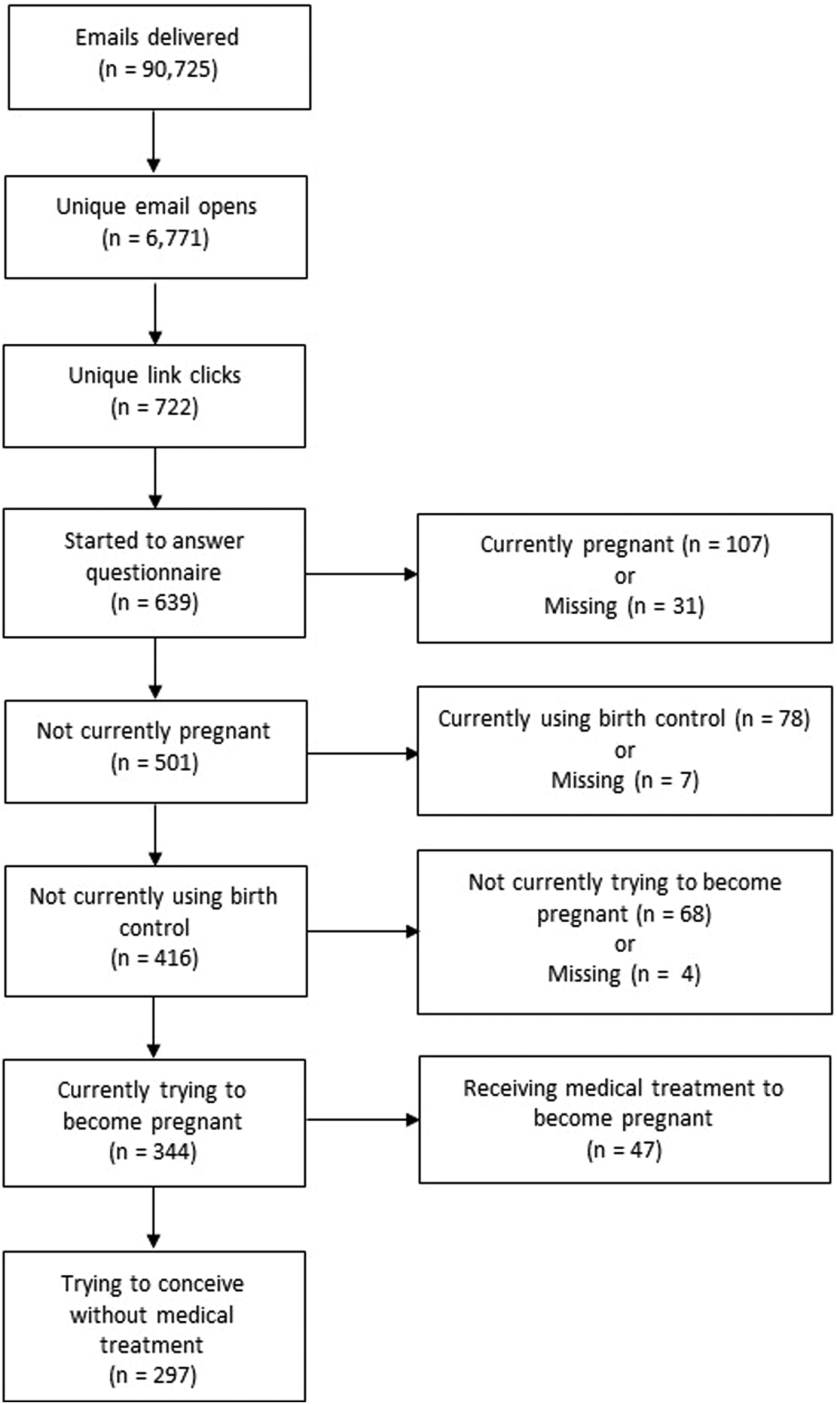
Flow chart of participant inclusion.

All respondents to the questionnaire answered simple demographic questions, such as age, race, ethnicity, and education (*N* = 639, Figure 1). Data from these respondents are used to describe the demographic characteristics of participants recruited from a cycle tracking app userbase (objective 1). In addition, Ovia Health collects demographic variables upon user enrollment, such as race, ethnicity, education level, and relationship status. These statistics reflect the composition of the Ovia Fertility userbase as of 2022. These aggregate statistics were compared to the demographics of these survey respondents.

The next series of questions identified those who were attempting to become pregnant (Figure 1). First, those who were currently pregnant were identified and excluded from the remainder of the questionnaire (*N* = 107). Second, participants were asked, “Are you currently using any form of birth control (including but not limited to, the pill, implant, IUD, condoms, abstinence, etc)?” if they said yes, they were considered not trying to become pregnant (*N* = 78). Third, those who were not pregnant and not using birth control were asked, “Are you currently trying to become pregnant? While there are many ways to become pregnant, in this case we are talking about currently having regular unprotected intercourse with a male partner.” If the participant answered “no” to this question they were considered not trying to conceive (*N* = 68). The latter two groups of participants were further asked, “Are you planning to try to become pregnant in the next 3 months?” and this was the last question they were asked. Data from those on birth control and those not currently trying were used to estimate the proportion of app users who plan to start a pregnancy attempt in the next 3 months and might then be eligible for enrollment in a time-to-pregnancy study (objective 2) (*N* = 146). Finally, respondents who reported attempting to become pregnant, were asked, “Are you currently receiving any medical treatments to help you get pregnant?” and if they answered yes, they were excluded from the remainder of the questionnaire (*N* = 47).

Additional questions were asked of those who were attempting to become pregnant without medical intervention (*N* = 297) to assess the diversity of their demographic and reproductive characteristics and to describe the current pregnancy attempt and the factors that affect pregnancy planning (objectives 3–6). The additional questions included pregnancy attempt duration, factors influencing pregnancy planning, reproductive and birth control history, and health history and behaviors while attempting to conceive. Body mass index was calculated from the reported height and weight. Users could select multiple factors when identifying influences on their pregnancy planning and their behaviors while attempting to conceive. Finally, they were asked to invite their partners to complete the partner portion of the questionnaire, which included similar domains to those previously described, including demographics and behaviors while attempting to conceive. The partner questions were at the end of the questionnaire that was sent to the app user. We characterized questionnaire responses with frequencies and percentages.

## Results

### Objective 1: Describe the demographic characteristics of all questionnaire respondents recruited through Ovia Fertility and compare with the Ovia Fertility userbase

In 12 days, a total of 90,725 emails were delivered to unique email addresses,6,771 were opened, and there were 722 unique link clicks (Figure 1). Of these, 639 people answered at least a portion of the survey. Of these, 107 were currently pregnant and 31 were missing pregnancy status, 78 were currently using birth control, and 68 were not currently trying to become pregnant. This left 344 participants who were trying to become pregnant of whom 47 were receiving medical treatments to become pregnant. The remaining 297 participants were trying to become pregnant without medical intervention and eligible for recruitment to a prospective time-to-pregnancy study.

The median time spent answering the survey was 4 min (25th, 75th percentiles: 1, 10 min). Among the 639 who responded to the demographics and descriptive portion of the survey, over 500 responded within 1 day, and 600 responded within 5 days. All respondents participated within 10 days of receiving the questionnaire. The majority of respondents were aged 26–35, self-reported their race as White and their ethnicity as non-Hispanic, had at least a bachelor's degree, were married, and most had an income of $50,000 to $200,000 (Table 1). Respondents came from 49 states and Washington DC, with the largest proportion from California (Table 1).

**Table 1:** Demographics of all survey respondents compared with respondents who were trying to conceive, and the Ovia Fertility U.S. userbase.

	Full sample (*n* = 639)		Trying to conceive (*n* = 297)		Ovia Fertility userbase (2022)
	*N*	(%)	*N*	(%)	%
Age					
18–25	59	10	29	10	11
26–30	167	27	85	29	21
31–35	215	35	102	34	37
36–40	118	19	51	17	25
>40	50	8	30	10	6
Missing	30		0		
Race					
White	443	73	208	70	64
Black/African American	69	11	40	13	14
Asian, Native American/Native Alaskan, or Native Hawaiian/other Pacific Islander	33	5	20	7	90
Hispanic^a^					10
Multiple races	27	4	15	5	3
Declined to respond	38	6	14	5	
Missing	29		0		
Ethnicity					
Hispanic/Latina	80	13	38	13	n/a^a^
Not Hispanic/Latina	521	86	258	86	
Missing	38		1		
Education					
Some high school, high school diploma or equivalent	53	8	32	11	41
Some college but no degree	94	15	46	16	^b^
Associate or technical degree	100	16	52	18	14
Bachelor's degree	205	34	102	34	28
Master's or doctoral degree	153	25	64	22	17
Missing	34		1		
Relationship status					
Single, never married	48	8	19	6	8
Committed relationship	85	14	54	18	33
Married or domestic partnership	461	76	220	74	58
Other or missing	45		4		1
Income					
Less than $20,000	41	7	20	7	^c^
$20,001 to $50,000	119	20	65	23	
$50,001 to $100,000	211	35	102	37	
$100,001 to $200,000	145	24	70	25	
More than $200,000	42	7	22	8	
Missing	81		18		
State^d^					
Arizona	21	4	11	4	^c^
California	66	11	31	11	
Florida	40	7	14	5	
Illinois	27	5	12	4	
Massachusetts	23	4	12	4	
New York	42	7	26	9	
North Carolina	20	3	15	5	
Ohio	23	4	10	3	
Pennsylvania	21	4	11	4	
Texas	35	6	15	5	
Other states	280	47	136	46	
Missing	41		4		

^a^ Race and Hispanic ethnicity were not captured as separate questions in the Ovia Fertility user database.

^b^ This category is not captured separately in the Ovia Fertility user database. App users with “some college” are classified as “high school diploma”.

^c^ Ovia Health's policy is not to publish income level and geography.

^d^ Ten most highly represented states.

In the Ovia Fertility userbase 60% report that they are trying to conceive while 40% report not trying to conceive. The proportion trying to conceive was similar in our survey (344/639 or 54%, Figure 1). Compared with the Ovia Fertility userbase, the survey respondents were slightly younger, had higher educational attainment and were more likely to be married (Table 1). A higher proportion of survey respondents reported their race as White (73%) compared with the Ovia Fertility userbase (64%), while a similar proportion reported their race as Black or African-American (11% vs. 14%) however, the Ovia Fertility userbase does not query race separately from Hispanic ethnicity, which limits the direct comparison of these proportions.

### Objective 2: Estimate the proportion of individuals using the app who plan to start a pregnancy attempt in the next 3 months, and would soon be eligible for enrollment in a time-to-pregnancy study

Of the 146 respondents who were not currently trying to conceive, 12% did plan to try in 3 months, 3% were unsure about trying in the next 3 months, 69% were not planning to start trying in the next 3 months and this decision was unrelated to COVID-19, and 16% of respondents were not planning to start trying or were unsure whether they would start trying at least partially due to COVID-19.

### Objective 3: Estimate the proportion of individuals who are currently attempting pregnancy without medical treatments and could therefore be immediately eligible for a time-to-pregnancy study

Of the 639 respondents, 344 (54%) were currently trying to become pregnant and of those, 297 (86%) were not using medical treatments to conceive (46% of the total sample).

### Objective 4: Among those who were currently attempting to conceive without medical interventions, assess the diversity of the demographic and reproductive characteristics and describe characteristics of their current pregnancy attempt

Demographic characteristics of those trying to conceive were similar to those of all of the survey respondents (Table 1). About one-third of the participants were of recommended BMI, 24% were overweight, and 41% were in obese categories (Table 2). A small proportion of respondents reported ever smoking (19%) and of these, few were current smokers (7%). About one-third of participants self-reported a history of infertility (Table 2). For almost 60% of participants, the longest attempt time was 3 months or less.

**Table 2:** Behavioral and reproductive characteristics of participants who were trying to conceive (*n* = 297).

	*n*	(%)
BMI		
Underweight or normal (<24.9)	87	35
Overweight (25.0–29.9)	60	24
Obese (>30.0)	102	41
Missing	48	
Ever smoked cigarettes on a regular basis		
Yes	46	19
No	202	81
Missing	49	
Currently smoke one or more cigarettes per day		
Yes	18	7
No	230	93
Missing	49	
Ever had regular unprotected intercourse for 12 months or more without becoming pregnant?		
Yes	91	37
No	157	63
Missing	49	
What is the longest amount of time it took to conceive any of your pregnancies?		
≤3 months	79	58
>3–6 months	19	14
>6–12 months	19	14
>12 months	19	14
Missing	161	3
Has a doctor or other health professional ever told you that you had any of the following conditions? (Please select all that apply)		
Polycystic ovary syndrome (PCOS)	15	5
Uterine fibroids	12	4
Pelvic inflammatory disease	1	0.3
Endometriosis	14	5
Irregular menstrual cycles	38	13
Autoimmune disease, such as lupus, Grave's disease, Sjogren's, multiple sclerosis	14	5
None of the above	173	58
Missing	48	
Ever been pregnant		
Yes	136	55
No	112	45
Missing	49	
How many times have you been pregnant?		
1	61	45
2	38	28
3	16	12
>3	21	15
Missing^a^	161	
How many of your pregnancies ended in a pregnancy loss?		
0	53	39
1	58	43
>1	25	18
Missing^a^	161	
How many of your pregnancies resulted in a live birth?^a^		
0	43	32
1	53	39
2	27	20
>2	13	10
Missing^a^	161	
Ever conceived a pregnancy while using birth control?		
Yes	22	16
No	114	84
Missing^a^	161	

^a^ Includes those who were never pregnant.

Most participants reported no history of health conditions related to fertility (58%). Irregular cycles had the highest prevalence (13%) (Table 2). Over half of the sample had been pregnant before (55%). Of those who had been pregnant, 85% had been pregnant 3 times or less, 39% had never had a pregnancy loss, and 43% had one previous loss.

Forty-eight participants (17%) had been trying to conceive for 1 month or less, 88 (31%) had been trying for 2 months or less, and 122 (43%) had been trying for 3 months or less. Among the 73 participants who reported a history of infertility but did not report an attempt time of 12 months or more for any of their pregnancies, 63% had been trying for at least a year in their current attempt, which indicates that the current attempt was the longest attempt in most cases. It is also possible that previous long attempts did not result in a pregnancy (Table 3).

**Table 3:** Characteristics of the current pregnancy attempt among those who were trying to conceive (*n* = 297).

	*N*	%
For how long have you been trying to become pregnant?		
1 month or less	48	17
>1 to 3 months	74	26
>3 to 6 months	46	16
>6 to 12 months	37	13
>12 to 24 months	47	17
>24 months	31	11
Missing	14	
Days since stopping any form of birth control^a^		
1 month or less	18	7
>1 to 3 months	50	20
>3 to 6 months	39	16
>6 to 12 months	39	16
>12 to 24 months	37	15
24 or more months	65	26
Missing	49	
How much time passed between the time you decided you wanted to be pregnant to the date you started trying to become pregnant?		
1 month or less	74	26
>1 to 3 months	46	16
>3 to 6 months	39	14
>6 to 12 months	51	18
>12 to 24 months	34	12
24 or more months	38	13
Missing	15	
What factors influenced when you decided to try to become pregnant? Please rank your top three:		
I was planning the pregnancy based on my age.	138	46
I wanted to deliver a baby at a certain time of year.	61	21
I wanted to be pregnant at a certain time of year.	28	9
I was planning the pregnancy around my job responsibilities.	75	25
I was planning the pregnancy around my education or schooling.	24	8
I was planning the pregnancy based on my access to insurance coverage or medical care.	16	5
I didn't want to plan, I just wanted to let it happen.	81	27
I didn't want to use or continue using birth control.	33	11
I was planning the pregnancy around my partner's job responsibilities.	16	5
I was planning the pregnancy around my partner's education or schooling.	10	3
Coronavirus (COVID-19) influenced my decision (Please explain)	15	5
Other (Please explain)	34	11
During this pregnancy attempt, have you been trying to become pregnant…		
The entire time, consistently	172	61
Most of the time, but with some birth control use or protected intercourse or sometimes my partner and I did not have intercourse	88	31
Most of the time, we paused trying because of COVID	17	6
Missing or other response	19	
Which birth control method (s) did you use in the 3 months **before you stopped using birth control? (Participants selected all that apply.)**		
Birth control pills	110	37
Hormonal implant (such as Norplant or Implanon), patch, or vaginal ring	12	4
Hormone shots like Depo-Provera	12	4
IUD	32	11
Male condom	68	23
Fertility awareness or calendar method	19	6
Abstinence	18	6
No birth control or withdrawal method	17	6
Other	27	9
Are you doing any of the following to improve your chances of pregnancy? Please select all that apply.		
Using ovulation predictor kits	117	39
Taking my temperature	58	20
Checking my cervical mucus	101	34
Exercising	111	37
Drinking fertility tea	12	4
Taking vitamins or nutritional supplements	153	52
Eating more or less of certain foods	49	17
Avoiding chemicals	23	8
Avoiding prescription medications	12	4
Avoiding over-the-counter medications	25	8
Other^b^	13	4
None of the above	27	9

^a^ Calculated from the date of the questionnaire to the date reported for this question: “On what date did you and your partner completely stop using any form of birth control? If you don't remember exactly, please provide your best guess.”

^b^ This category includes several items such as, “aromatherapy”, “essential oils”, or “acupuncture or acupressure”.

One-quarter had stopped birth control within the past 3 months (27%), about one-third had stopped 3–12 months prior (31%), and 41% had stopped more than 12 months prior (Table 3).

A little more than half (52%) reported 6 months or more between deciding to become pregnant and starting to try, however, the most common response was 1 month or less (26%) (Table 3). The median time for women under 30 was 243 days (25th, 75th percentiles: 36, 403) which was longer than the time for women 30 and over, 137 days (25th, 75th percentiles: 30, 365). The median time for those with a high school education or less was 198 days (25th, 75th percentiles: 30, 730) which was slightly longer than those with a masters or doctoral degree, 122 days (25th, 75th percentiles: 30, 365). Participants who reported their race as White reported a median time of 122 days (25th, 75th percentiles: 30, 365) while those who reported their race as Black or African American reported a median time of 365 days (25th, 75th percentiles: 91, 730).

Over one-third of participants reported interruptions in their pregnancy attempt (37%), 31% due to periods of time without intercourse, and 6% due to COVID19 (Table 3).

Respondents to our questionnaire reported a high level of engagement with the app, using it daily (44%) or multiple times per week (29%) (Table 4).

**Table 4:** Engagement with the app among those who were trying to conceive (*n* = 297).

	Median (IQR)	
Time since first started using app (weeks)	26 (8.5, 87)	
Missing: *n* = 31, 10.44%		
	*n*	(%)
How often do you use the app?		
More than once a day	27	10
Daily	117	44
Multiple times each week	78	29
Only once a week	14	5
Multiple times each month or less	31	12
Missing	30	
What do you use the Ovia Fertility app for? (Please select all that apply)		
Improving the chances of pregnancy	214	71
Tracking menstrual periods	231	76
Timing intercourse	188	62
Tracking ovulation	221	73
Fertility advice	133	44
Missing	30	

### Objective 5: Among those who were currently attempting to conceive without medical intervention we aimed to describe the factors that influence app users’ decisions around pregnancy planning

The most frequently endorsed factor that influenced starting to try was age (46%) (Table 3). The next most frequent responses were “I didn't want to plan, I just wanted to let it happen” (27%), “I was planning the pregnancy around my job responsibilities” (25%), or “I wanted to deliver a baby at a certain time of year” (21%). Among women less than 30 years of age, age was still the most commonly reported factor (39%), however, younger women were more likely than older women to report that they “just wanted to let it happen,” (33% vs. 25%) (Supplementary Table S1). Among participants who reported their race as Black or African American the most common response was wanting to “let it happen” (48%) and then age (37%). Among those with a high school diploma or less, the most common response was “let it happen” and this was far more common in this group than in other education groups (62% vs. 11%–39%). Wanting to “let it happen” was more commonly reported among those who reported a BMI consistent with obesity (35%). Finally, respondents from the western U.S. were least likely to report wanting to “let it happen” (19%) compared with other regions (25%–34%). Respondents in the Midwest (27%) and West (28%) were more likely to report wanting to deliver a baby at a certain time of year. Participants from the South were more likely to report not wanting to use birth control (17%) compared with other regions (7%–9%).

The most frequently selected items were, “taking vitamins or nutritional supplements,” (52%), “using ovulation predictor kits,” (39%), “exercising,” (37%), and “checking my cervical mucus,” (34%) (Table 3). About 9% selected “none of the above”. The amount of time trying was shorter for those who selected “none of the above” (*N* = 27, median = 60 days, 56% trying less than 90 days) compared with those who reported at least one behavior (*N* = 222, median = 152 days, 31% trying less than 90 days).

### Objective 6: Among those who were currently attempting to conceive without medical intervention, assess the willingness of partners to participate in a research study

Nearly half (47%) of partners agreed to participate in the survey (Table 5). The characteristics of partners mirrored the characteristics of respondents, although they were slightly older (Table 5). The most frequently selected behavior was “exercising” (53%), followed by “taking vitamins or nutritional supplements” (26%). About one-third (36%) reported not doing any of the behaviors listed.

**Table 5:** Partner characteristics (*n* = 117).

	*n*	(%)
Partner willing to participate in this kind of questionnaire?^a^		
Yes	117	47
No	130	53
Missing	50	
Age		
18–25	10	9
26–30	28	24
31–35	33	28
36–40	32	27
>40	14	12
Race		
White	81	74
Black/African American	15	14
Other	14	13
Missing	7	
Ethnicity		
Hispanic/Latino	18	16
Not Hispanic/Latino	97	84
Missing	2	
Education		
Some high school, high school diploma, or equivalent	22	19
Some college but no degree	28	24
Associate or technical degree	17	15
Bachelor's degree	30	26
Master's or doctoral degree	19	16
Missing	1	
BMI		
Normal (18.5–24.9)	36	31
Overweight (25.0–29.9)	45	39
Obese (>30.0)	35	30
Missing	1	
What are you doing to improve your partner's chances of pregnancy? Select all that apply.		
Exercising	62	53
Taking vitamins or nutritional supplements	31	26
Eating more or less of certain foods	14	12
None of the above	42	36

^a^ For this row only, *n* = 297.

## Discussion

We aimed to describe the cycle tracking app users who were interested in participating in pre-pregnancy research studies. In a recruitment period of less than 2 weeks, over 90,000 emails were delivered to Ovia Fertility users. This resulted in a total of 639 respondents of whom 297 respondents reported actively trying to conceive without using a medical intervention. Most questionnaires were completed within a few days of the email being sent. This descriptive analysis is the first of its kind to describe the characteristics of cycle tracking app users likely to be recruited for prospective time-to-pregnancy studies or for preconception pregnancy studies.

Like previously described volunteer, time-to-pregnancy studies (5–8), participants recruited for this study through Ovia Fertility tended to have at least a college degree and identified their race as White. However, our survey respondents were diverse with respect to income, geographic location, and body mass index. To increase representation, an invitation might be targeted to historically underrepresented racial or ethnic groups if the app has collected those data. Also, our study sent a single invitation email to each address and additional follow-up emails could be sent especially to groups with lower initial response rates.

Most participants reported that they had no history of health conditions related to fertility. The prevalence of any one of the queried conditions that might reduce fertility (PCOS, endometriosis, or uterine fibroids) among respondents was around 5%, which is the same or lower than most population estimates (9). The prevalence of irregular cycles in this sample was approximately 13%, which is similar to the 10% reported in the Nurses’ Health Study II (10) but lower than a European cohort which reported 19% (11). This suggests that people with diagnosed conditions affecting fertility would not be disproportionately recruited from app users.

A high proportion of respondents trying to conceive self-reported a history of pregnancy loss (61%), which might indicate that people begin using an app after experiencing a pregnancy loss. Additionally, a substantial proportion of respondents reported a history of infertility (37%). The current pregnancy attempt was longer than 12 months for 28% of the sample, but 43% of respondents had been trying to conceive for 3 months or less. This distribution is somewhat shifted to longer attempt times compared with other time-to-pregnancy cohorts. One previous cohort reported 56% had been trying to conceive for less than 3 months at enrollment and 9% for at least 12 months (12). Another study reported a median attempt time of 3 months at enrollment (in our study it was about 5 months) (13). This may be partially explained by length-biased sampling (14) which occurs when people are recruited to a study after their pregnancy attempt has begun. To address this selection bias and reduce inclusion of infertile participants, time-to-pregnancy studies may choose to limit study enrollment to those who have not previously experienced infertility and enroll app users who have only been trying for a short time, such as less than 30, 60, or 90 days, or even limit to women planning to start trying in the near future.

The distribution of attempt time was shorter, overall, than the distribution of the time since stopping birth control. This could be due to many things such as a prior pregnancy followed by a period without birth control before trying again, a waiting period after hormonal birth control, or a change in family situation. There may also have been women who were having sex and not preventing pregnancy, but it was only later that they considered themselves to be “trying”. Future queries will be needed to understand the reasons for the differences between these two responses and to develop questions that can be used to assess this interesting phenomenon in future time to pregnancy studies.

Respondents reported doing several things to improve their chances of pregnancy including taking vitamins, using ovulation predictor kits and cervical mucus monitoring, exercising, and eating more or less of different foods. Further research on the effects of these behaviors on time to pregnancy would thus be feasible, given their prevalence. On the other hand, behaviors such as aromatherapy, essential oils, acupuncture/acupressure were rare and would be more difficult to research. The amount of time trying was shorter for those who reported not doing any of the behaviors listed compared with those who did report at least one behavior listed. This likely indicates that couples are more likely to add or change their behaviors to try to increase the chance of conception as their pregnancy attempt lengthens, which has been reported previously (15). It will be helpful in prospective time-to-pregnancy studies to regularly ask, potentially at the beginning of each menstrual cycle, what the couple might be doing to help them conceive and to include a response option of just “letting it happen”.

Among those who were currently using birth control or were not trying to become pregnant, 12% were planning to start a pregnancy attempt in the next 3 months and another 15% were unsure. Among those who were trying to conceive, we asked the amount of time between deciding to become pregnant and starting to try. This question characterizes the amount of time a person knows they are going to try to conceive but hasn't yet started their attempt. The longer this time window is, the more likely a time-to-pregnancy study could find and invite this person to pre-enroll in the study before the attempt begins. This would allow for monthly follow-up of pregnancy prevention methods and unprotected intercourse events so that variation in what is meant by “starting to try to conceive” can be described. Targeting and pre-enrolling app users who are planning to begin a pregnancy attempt may be a special advantage of app-based recruitment because it would allow for more precision in identifying the true start of a pregnancy attempt. Our data indicated that for one quarter of the respondents the planning window that could be targeted for pre-enrollment is quite short, 1 month or less. Another 30% reported a planning window of 1–6 months, and about 44% of the respondents reported a window of more than 6 months. In addition, although the sample was small, we found some evidence that this time is more than twice as long in Black or African American participants compared with White participants. Differences by age and education were much smaller.

When recruiting participants for a prospective preconception study, it is useful to recognize the influences on timing the start of a pregnancy attempt. Age was the most frequently reported influence, but timing of the year was also important, indicating that there may be seasonal patterns to pregnancy attempts (16). This may influence selection and recruitment efficiency across the year. A substantial proportion of respondents also reported wanting to “let it happen.” We are unaware of any data on how these factors that affect timing of an attempt might also influence willingness to participate in a research study or the subsequent quality of data collection for those who do enroll, for example, does wanting to “let it happen” influence a person's willingness to enroll in a detailed prospective study? While the sample of Black and African American participants was small, there was some evidence that wanted to “let it happen” was the most common motivation for starting a pregnancy attempt and was even more frequently reported than age. Further research on factors that influence pregnancy attempt timing would be valuable.

Our data show that 40% of respondents experienced interruptions in their attempt to become pregnant. Despite the survey being deployed during the COVID19 pandemic, most interruptions were unrelated to the pandemic and were instead related to other factors, such as employment, preference, or other reasons. This highlights the importance of collecting information regarding these interruptions during a prospective study, which is supported by a previous study that examined women's cycle-to-cycle variability in pregnancy intention (17). The frequency and length of the interruptions may be related to other demographic or behavioral variables that are being examined for their association with time to pregnancy. Unaccounted for interruptions will contribute to measurement error, and if errors differ by exposure status, the estimates will be biased.

About half of the partners of respondents were willing to participate in this research questionnaire. The importance of partners for time to pregnancy research has been increasingly recognized (18). Further research on partners’ motivation for joining a research study is important for improving participation rates. Of the partners that responded to our survey, about half reported exercising and one quarter taking supplements to improve their partner's chances of pregnancy. These data indicate that research on these behaviors might have adequate sample size.

In summary, recruiting menstrual tracking app users for prospective time-to-pregnancy studies could have several advantages. Cycle tracking mobile applications have become a popular tool for millions of individuals to track and better understand their reproductive health. These applications generate massive datasets related to fertility, menstrual cycle data collection, and other reproductive health indicators. Time-to-pregnancy studies can be limited by small sample sizes and are time-consuming to enroll. Recruitment for time-to-conception or pregnancy research through cycle tracking apps can be rapid and geographically diverse. The large base population of users can allow for stratified study enrollment based on specific factors such as race and ethnicity so that inclusivity of enrollment is assured.

## Data Availability

The data generated for this study can be obtained by written request from the corresponding author.

## Supplementary material

Supplementary Table 1 Factors that influenced the timing of a pregnancy attempt stratified by participant characteristics.

Supplementary Image 1 Recruitment email sent to selected Ovia Fertility app users.
