# Supplementary material for: Feasibility of leveraging menstrual cycle tracking apps for preconception research recruitment
Source: Front Reprod Health. 2022 Sep 30;4:981878. doi: 10.3389/frph.2022.981878 (PMC9580765; doi:10.3389/frph.2022.981878)
Supplement: Supplementary Table 1 Factors that influenced the timing of a pregnancy attempt stratified by participant characteristics. [file Table1.docx]

| Supplemental Table 1: Factors that influenced the timing of a pregnancy attempt stratified by participant characteristics. | | | | | | | | | | | | | | | | | | |
| --- | --- | --- | --- | --- | --- | --- | --- | --- | --- | --- | --- | --- | --- | --- | --- | --- | --- | --- |
| Characteristic |  |  |  | What factors influenced when you decided to try to become pregnant? ^1^ | | | | | | | | | | | | | |  |
|  |  | Overall |  | Based on my age | |  | Just let it happen | |  | Job Duties | |  | Deliver at a time of year | |  | Didn’t want birth control | | |
|  |  | n |  | n | (%) |  | n | (%) |  | n | (%) |  | n | (%) |  | n | (%) | |
| N |  | 297 |  | 138 | 46 |  | 81 | 27 |  | 75 | 25 |  | 61 | 21 |  | 33 | 11 | |
| Age |  |  |  |  |  |  |  |  |  |  |  |  |  |  |  |  |  | |
| <30 |  | 94 |  | 37 | 39 |  | 31 | 33 |  | 23 | 24 |  | 23 | 24 |  | 16 | 17 | |
| >=30 |  | 203 |  | 101 | 50 |  | 50 | 25 |  | 52 | 26 |  | 38 | 19 |  | 17 | 8 | |
| Race |  |  |  |  |  |  |  |  |  |  |  |  |  |  |  |  |  | |
| NA, A, H, Asian, PI, or multiple^2^ |  | 35 |  | 16 | 46 |  | 10 | 29 |  | 8 | 23 |  | 8 | 23 |  | 3 | 9 | |
| Black/African American |  | 40 |  | 15 | 37 |  | 19 | 48 |  | 5 | 12 |  | 7 | 18 |  | 6 | 15 | |
| White |  | 208 |  | 99 | 48 |  | 47 | 23 |  | 58 | 28 |  | 45 | 22 |  | 22 | 11 | |
| Missing |  | 14 |  |  |  |  |  |  |  |  |  |  |  |  |  |  |  | |
| Ethnicity |  |  |  |  |  |  |  |  |  |  |  |  |  |  |  |  |  | |
| Hispanic/Latina |  | 38 |  | 15 | 40 |  | 11 | 29 |  | 10 | 26 |  | 8 | 21 |  | 4 | 10 | |
| Not Hispanic/Latina |  | 258 |  | 123 | 48 |  | 69 | 27 |  | 65 | 25 |  | 53 | 20 |  | 29 | 11 | |
| Education |  |  |  |  |  |  |  |  |  |  |  |  |  |  |  |  |  | |
| High school diploma or less |  | 32 |  | 9 | 28 |  | 20 | 62 |  | 3 | 9 |  | 5 | 16 |  | 6 | 19 | |
| Some college |  | 46 |  | 20 | 43 |  | 17 | 37 |  | 3 | 7 |  | 7 | 15 |  | 5 | 11 | |
| Associate/technical |  | 52 |  | 25 | 48 |  | 20 | 39 |  | 6 | 11 |  | 9 | 17 |  | 3 | 6 | |
| Bachelor's degree |  | 102 |  | 50 | 49 |  | 17 | 17 |  | 29 | 28 |  | 26 | 25 |  | 12 | 12 | |
| Master's or doctoral |  | 64 |  | 34 | 53 |  | 7 | 11 |  | 34 | 53 |  | 14 | 22 |  | 7 | 11 | |
| Missing |  |  |  |  |  |  |  |  |  |  |  |  |  |  |  |  |  | |
| BMI |  |  |  |  |  |  |  |  |  |  |  |  |  |  |  |  |  | |
| Non-obese |  | 147 |  | 74 | 50 |  | 34 | 23 |  | 41 | 28 |  | 35 | 24 |  | 18 | 12 | |
| Obese |  | 102 |  | 48 | 47 |  | 36 | 35 |  | 27 | 27 |  | 20 | 20 |  | 14 | 14 | |
| Missing |  | 48 |  |  |  |  |  |  |  |  |  |  |  |  |  |  |  | |
| Region^3^ |  |  |  |  |  |  |  |  |  |  |  |  |  |  |  |  |  | |
| Midwest |  | 60 |  | 30 | 50 |  | 18 | 30 |  | 16 | 27 |  | 16 | 27 |  | 4 | 7 | |
| Northeast |  | 59 |  | 27 | 46 |  | 15 | 25 |  | 15 | 25 |  | 9 | 15 |  | 5 | 9 | |
| South |  | 100 |  | 49 | 49 |  | 34 | 34 |  | 21 | 21 |  | 12 | 12 |  | 17 | 17 | |
| West |  | 74 |  | 31 | 42 |  | 14 | 19 |  | 19 | 26 |  | 21 | 28 |  | 7 | 9 | |
| Missing |  | 4 |  |  |  |  |  |  |  |  |  |  |  |  |  |  |  | |
| 1. Participants could select multiple reasons for trying; each column shows demographics among participants who listed each reason. The full text for each response option were: “I was planning the pregnancy based on my age”, “I didn’t want to plan, I just wanted to let it happen”, “I was planning the pregnancy around my job responsibilities”, “I wanted to deliver a baby at a certain time of year”, and “I didn’t want to use or continue birth control”. | | | | | | | | | | | | | | | | | | |
| 2. Native American, Alaskan, Hawaiian, Asian, Pacific Islander, or multiple races. | | | | | | | | | | | | | | | | | | |
| 3. Census Regions of the United States. | | | | | | | | | | | | | | | | | | |
|  | | | | | | | | | | | | | | | | | | |
