# Supplementary figures and images for: Feasibility of leveraging menstrual cycle tracking apps for preconception research recruitment
Source: Front Reprod Health. 2022 Sep 30;4:981878. doi: 10.3389/frph.2022.981878 (PMC9580765; doi:10.3389/frph.2022.981878)

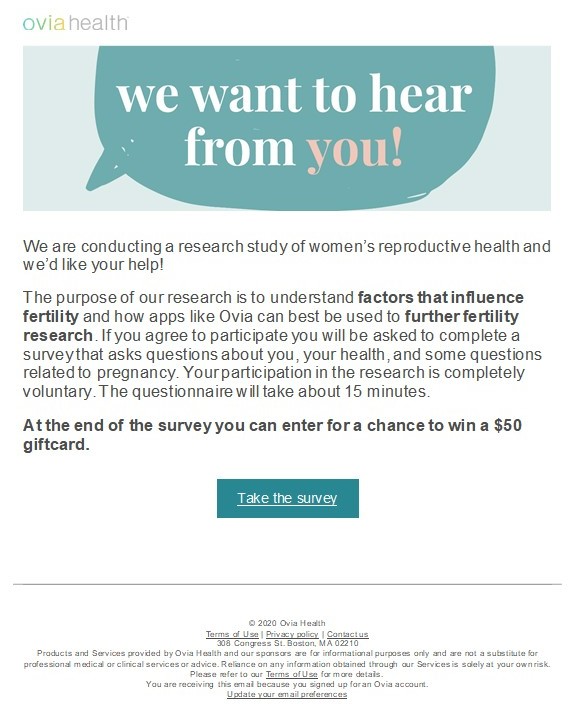

Supplement: Supplementary Image 1 Recruitment email sent to selected Ovia Fertility app users. [file Image1.jpeg]
